# Three Licorice Extracts’ Impact on the Quality of Fresh-Cut Sweet Potato (*Ipomoea batatas* (L.) Lam) Slices

**DOI:** 10.3390/foods13020211

**Published:** 2024-01-09

**Authors:** Ximing Xu, Heyao Zhang, Sheng Jin, Yueming Zhu, Zunfu Lv, Peng Cui, Guoquan Lu

**Affiliations:** The Key Laboratory for Quality Improvement of Agricultural Products of Zhejiang Province, Institute of Root and Tuber Crops, College of Advanced Agricultural Sciences, Zhejiang A&F University, Hangzhou 311300, China; xuximing@zafu.edu.cn (X.X.); zhyao@stu.zafu.edu.cn (H.Z.); zhuym@zafu.edu.cn (Y.Z.); lvzunfu@163.com (Z.L.); cuipeng626@163.com (P.C.)

**Keywords:** licorice extracts, fresh-cut sweet potato slices, texture properties, appearance properties, antioxidant capacities

## Abstract

The quality of fresh-cut produce, particularly sweet potatoes, is crucial for their value. Licorice extract is an optional additive in fresh-cut sweet potatoes. This study examined the impact of three licorice extracts (licorice acid, LA; licorice flavonoids, LF; and licorice polysaccharides, LP) on the quality of fresh-cut sweet potato slices (FCSPSs) for one week of storage. After one week of storage, the extracts showed varying effects on FCSPSs. LA and LF treatments reduced the area proportion of browning (APB), while LP treatments increased APB and decreased L* values. Antioxidant experiments revealed that LP treatments increased PPO and POD activity while reducing SOD activity. The concentrations of the three licorice extracts showed a strong negative correlation with SOD activity. In conclusion, LP harmed the appearance and antioxidant qualities of FCSPSs. LA and LF may be suitable additive components for FCSPSs, and 30 mg/mL LA and LF treatments were found to maintain the appearance and texture quality of FCSPSs during storage. Therefore, careful consideration should be given when using LP as a food additive for FCSPSs.

## 1. Introduction

The demand for fresh vegetables and fruits among modern people is increasing. Fresh-cut produce became popular because of high nutrition, freshness, cleanliness, convenience, safety, and attractiveness [1,2]. Nevertheless, ensuring the excellence of fresh-cut fruits and vegetables is a challenging endeavor [3]. Therefore, how to control the quality of fresh-cut produce in shelf life is an urgent problem for the growth of the fresh-cut produce industry. Sweet potato (*Ipomoea batatas* (L.) Lam) is a highly adaptable and widely grown crop that is cultivated in nearly 100 nations [4]. The Centre for Science in the Public Interest (CSPI) regards sweet potato as a ‘Super-food’ because it is abundant in nutrients, containing a substantial amount of carbs, dietary fiber, anthocyanins, β-Carotene, and vitamins. This versatile vegetable can be utilized in its raw form to create a variety of high-value products [5,6]. Sweet potatoes tend to undergo rapid oxidation and browning, leading to a decrease in quality [7]. Browning, which occurs due to both non-enzymatic and enzymatic reactions, is the primary issue in the processing of sweet potatoes [8]. Eating fresh-cut sweet potatoes has become more popular with the rise in the weight loss movement in recent years [3,6]. Nevertheless, the thin outer layer, abundant moisture, and pulpy underground organ of the sweet potato pose challenges when it comes to post-cut storage. They cause sweet potatoes to be easily wounded in the process of harvesting, packaging, storage, and processing. Wounded sweet potatoes brown and dry easily, and texture properties change. Hence, the development of fresh-cut sweet potatoes greatly relies on the importance of suitable food additive technology.

Food additives can be a viable option to maintain the quality of fresh-cut sweet potatoes during storage. However, chemical additives may cause environmental protection and food safety issues [9]. Consumers prefer fresh food without additives or with natural additives that are not harmful to health [10]. Licorice extracts are well-recognized for their antioxidant and antimicrobial activity, and they can be used in food [11]. Zhang et al. [12] found the mixture of licorice and rosemary extracts was a good food additive for meat due to it having strong antimicrobial activity against *Listeria monocytogenes*, *E. coli*, *P. fluorescens* and *L. sake*. Chakotiya et al. [13] found licorice extracts inhibited the growth of *Pseudomonas aeruginosa* on meat. Dietary licorice extracts improved antioxidant activity and protected meat against lipid oxidation [14]. Jiang et al. found licorice extracts had strong antioxidant activity to inhibit rancidity production [15]. Licorice extracts can hinder the invasion of *Helicobacter pylori* into the human body [16,17]. Licorice flavonoids, such as glabridin, lic-oricidin [18,19], and isoliquiritigenin [20], have good antioxidant activity. It protects the food against oxidation. These studies have given us new ideas for natural food additives for fresh-cut sweet potatoes.

Previous studies on the food additives of licorice have mostly focused on mixtures, with little focus on the main licorice extracts that affected FCSPSs. This study aims to realize three licorice extract treatments that affected FCSPSs. In this investigation, we formulated three primary pre-soaking techniques with licorice extract to produce consumable-quality FCSPSs. The outcomes of these treatments on the texture characteristics, visual attributes, and antioxidant capabilities of the FCSPS are detailed in this report.

## 2. Materials and Methods

### 2.1. Materials and Experiment Design

‘Xinxiang’ (a famous and popular edible sweet potato variety in China) was harvested from Zhejiang A & F University Agronomy Teaching practice base in 2022. Fresh and healthy sweet potatoes were harvested from farmland and transported to a laboratory within 24 h. Sweet potatoes were selected with 150–200 g weight and without surface damage. The fresh root samples were washed under running tap water and rinsed with sterile water, then air-dried inside the laboratory room for 2 h prior to storage. Slicers were used to cut the sweet potatoes into slices that were about 0.5 ± 0.1 cm. Then, we designed the control treatment (CK) and three licorice solution groups, groups A, B, and C, as shown in Table 1. The slices were dipped in three solution groups with concentrations of 10, 30, and 50 mg/mL for 10 min.

Licorice acid (LA, Glycyrrhizic acid, CAS 1405-86-3), licorice flavonoids (LF, Glabridin, CAS 59870-68-7), and licorice polysaccharides (LP, Liguiritigenin-7-O-D-apiosyl-4′-O-D-glucoside, CAS 199796-12-8) were purchased from Huiquan Biotechnology Co., Ltd. (Hangzhou, China). Thirty slices were placed onto a filter paper that was positioned inside every box. After sealing the boxes, they were placed for 7 days in dark environments in an incubator (PGX-500 C, Ningbo Brand Instrument Co., Ltd., Ningbo, China) that was adjusted to a temperature of 4 °C and humidity of 80%. Each treatment was replicated three times.

### 2.2. Textural Properties

The texture properties of FCSPSs were analyzed by a texture analyzer (TMS-PRO, FTC company, Washington, DC, USA) based on the techniques described in previous studies [6]. The TPA test was performed on the equatorial part of the disc using a physical proper-ty analyzer P/5 cylindrical probe (diameter 5 mm). Sections from different treatment groups were taken at 0, 1, 3, 5, and 7 d of storage and tested for TPA at room temperature under light conditions; each treatment’s sample was measured in 3 replicates. The definition of the parameters was measured by a texture analyzer.

### 2.3. Weight Loss and Decay Rate

The weight loss rate and decay rate of fresh-cut samples were measured according to Jiang [21]. We investigated the weight loss rate of FCSPS to acquire FCSPS from each group and weighed them during each test. The weight loss rate was calculated as follows:

$$Weight\;loss\;rate(\%)=\frac{{FCSPSs}^{’}\;quality\;on\;day\;0-{FCSPSs}^{’}\;quality\;at\;storage\;time}{{FCSPSs}^{’}\;quality\;on\;day\;0}\times 100$$

We also measured the rate and size of the decayed area of the FCSPS. The decay rate was calculated, and FCSPS with a spot width exceeding 5 mm were regarded as rotten FCSPS. The decay rate was evaluated as follows:

$$Decay\;rate(\%)=\frac{Number\;of\;rotten\;FCSPS}{Total\;number\;of\;FCSPS}\times 100$$

### 2.4. Appearance Quality

During sampling, the appearance of the fresh-cut sweet potato slices was photographed, and the environment and equipment were consistent each time. The area proportion of browning (APB) of slices was measured by Adobe Photoshop CS 5. We measured 3 groups for each treatment. The APB was evaluated as follows:

$$APB(\%)=\frac{Browning\;area\;of\;FCSPS}{Total\;area\;of\;FCSPS}\times 100$$

The flesh color of slices was measured according to Lin [22]. The CR-400 chroma meter (Konica Minolta Sensing Inc., Chiyoda, Japan) was used in this study. The color purity of FCSPS was evaluated as follows:

C* = (L*^2^ +a*^2^ + b*^2^)^0.5^.

“C*” indicates the color difference of FCSPS; “L” indicates the luster of the sample, with positive values indicating proximity to white and negative values indicating black. Similarly, “a*” indicates red or green, with positive values indicating a proximity to red and negative values indicating green. Lastly, “b*” indicates positive values indicating proximity to yellow and negative values indicating blue.

The degree of browning in the FCSPSs was determined by the extinction value method according to the method described by Liu et al. [23]. In total, 1.00 g of frozen sample was ground into a homogenate using 10 mL of ethanol. It was kept away from light for 30 min and stirred every 5 min. After centrifugation, the supernatant was obtained. The absorbance values were measured at 420 nm. The degree of browning of the FCSPS was evaluated as follows:

Browning degree value = A420 nm × 10 on a fresh weight basis.

### 2.5. Polyphenol Oxidase and Peroxidase Activities

The polyphenol oxidase (PPO) and peroxidase (POD) activity of fresh-cut samples were determined according to the method of Cao et al. [24]: 1.00 g of sample was ground into a homogenate using 5 mL of extraction buffer (0.05 mol/L pH 6.8 phosphate buffer) under an ice bath. Then, the sample was centrifuged at 12,000 rpm for 30 min at 4 °C. The supernatant was collected as the enzyme extract of PPO and POD. The PPO activity was measured by mixing 100 μL of the supernatant, and 4.0 mL of sodium acetate buffer solution (50 mmol/L, pH 5.5) with 1.0 mL of 50 mmol/L catechol solution, and the absorbance value was measured at 420 nm. The POD activity was measured by mixing 0.5 mL of supernatant and 3.0 mL of 25 mmol/L guaiacols with 200 μL of 0.5 mol/L hydrogen peroxide and measuring the absorbance at 470 nm.

### 2.6. Superoxide Dismutase Activities

The superoxide dismutase (SOD) activities of the samples were determined according to the method of Yu et al. [25]. In total, 1.00 g of the sample was ground into a homogenate using 5 mL of extraction buffer (PBS) under an ice bath. Then, the sample was centrifuged at 12,000 rpm for 10 min at 4 °C. The supernatant was collected as the enzyme extract of SOD. The supernatant was added to the reaction mixture, including 50 mM sodium phosphate buffer (pH 7.8), 0.75 mM nitroblue tetrazolium (NBT), 26 mM methionine, 0.02 mM riboflavin, and 1 μM ethylenediaminetetraacetic acid (EDTA). The absorbance was measured at 560 nm. The enzyme unit inhibited 50% of the NBT photochemical reduction.

### 2.7. Statistical Analysis

We followed the statistical analysis method used in our previous study [6]. The SPSS 20.0 software (IBM, Chicago, IL, USA) was used for the analysis of variance (ANOVA) and Scott-Knott cluster analysis. Origin 2021 (OriginLab, Northampton, MA, USA) was used for the mapping and correlation analysis. Values within the same storage day followed by the same letter are not significantly different at the 5% level according to ANOVA–Duncan’s multiple range test.

## 3. Results

### 3.1. Texture Properties

Texture properties are meaningful for sweet potatoes’ quality [26]. We conducted dynamic monitoring of the texture properties of FCSPSs, which were treated with three licorice extracts. Firmness is an intuitive indicator of FCSPSs. As shown in Table 2, the firmness of LP30 (96.26 ± 3.18 N) and LP50 (99.08 ± 4.59 N) was significantly higher than CK (84.50 ± 5.82 N, *p* < 0.05) at 1 day after storage (DAS). Other treatments were not significantly different from CK at 1 DAS (*p* > 0.05). At 3 DAS, the firmness of the FCSPSs after licorice solution treatment was higher than CK (75.96 ± 1.58 N), except for LA10 and LA30. The firmness of FCSPSs with LF and LP treatments was higher than CK significantly at 5 DAS (*p* < 0.05), and the LA-treated FCSPSs were not different from CK at 5 DAS (*p >* 0.05). The firmness of all the licorice solution treatments (82.72~99.24 N) was higher than CK (71.91 ± 5.08 N) at 7 DAS. This indicates that the three kinds of licorice solution treatments can increase the FCSPSs’ firmness 1 week after storage. The firmness of the FCSPSs after 30 and 50 mg/mL LP processing was still higher than CK (*p* < 0.05).

As shown in Table 3, cohesion of the FCSPSs after LA and LP treatments was significantly higher than CK at 1 DAS. The cohesion of the FCSPSs after LA and LP treatments was significantly higher than CK at 1 DAS. The cohesion of the FCSPSs after LA50, LF, and LP treatments was significantly higher than CK at 3 DAS. Only the cohesion of the FCSPSs after LF10 was significantly higher than CK at 5 DAS. The cohesion of the FCSPSs after LA10, LA50, and LF50 was higher than CK at 7 DAS. In total, we found the cohesion of FCSPSs after all licorice extract solutions treatments kept a higher value than CK in a week, although the value decreased partially over time. The cohesion of FCSPSs after LP treatments was highest at DAS 1 and 3, and then it was not significantly different from CK at DAS 5 and 7. It indicates that the effect of LP on the cohesion of the FCSPSs was only for 72 h. Partial treatments maintained FCSPSs and kept higher cohesion than CK in a week, such as LF. There was no significant difference with CK at DAS 1, but it kept a higher cohesion at DAS 3 and 7, which indicates this treatment. This indicates that the effect of this treatment on cohesion occurred after 24 h storage.

As shown in Table 4, gumminess of FCSPSs of CK (8.39~9.87 N) was lower than that of the three licorice extract solutions after the processing treatment during 1~7 DAS, except LA30 at 3 DAS (9.42 ± 1.69 N). All licorice treatments increased the gumminess of the FCSPSs significantly at 1 DAS. Nevertheless, this trend cannot be stably maintained in every treatment for a week, except LF30, LF50, and LP50. The gumminess of the FCSPSs after LF30, LF50, and LP50 processing was still higher than CK during 1~7 DAS. This indicates that the high-concentration LF and LP treatments were beneficial for the growth of the gumminess of the FCSPSs during storage.

As shown in Table 5, chewiness of the FCSPSs determines the palatability of FCSPSs. The chewiness of the FCSPSs after LA treatments was significantly higher than CK at DAS 1 and 5; however, they were not significantly different from CK at DAS 3. LA10 and LA50 were significantly higher than CK at DAS 7. The chewiness of the FCSPSs after LF treatments is still significantly higher than CK at storage time, except LF10 at 7 DAS. The chewiness of the FCSPSs after LP solution processing was higher than CK at 1~3 DAS, and then it decreased. LP10 treatment was significantly lower than CK at 7 DAS. This indicates that LP solution treatments significantly increase the chewiness of FCSPSs before day 3 of storage, while the trend was not sustained at 5~7 DAS.

### 3.2. Appearance Quality

**Figure 1:**
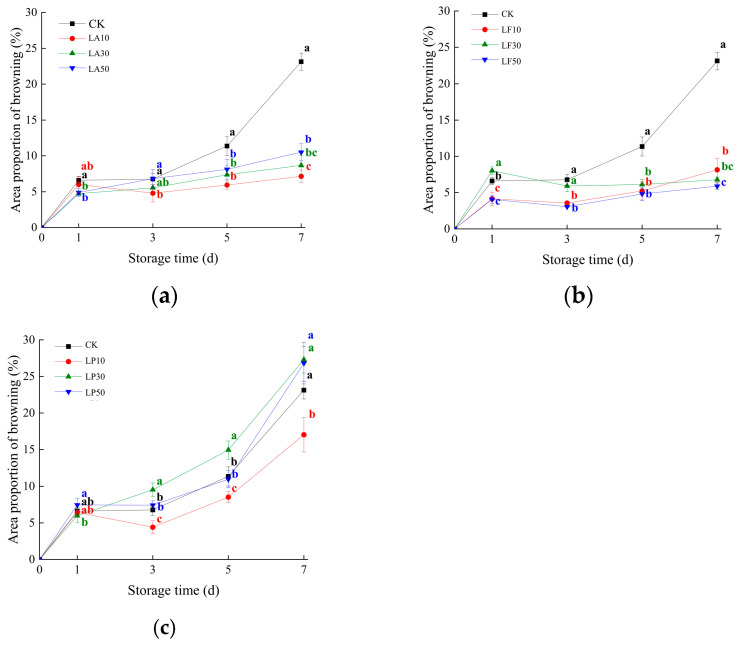
Area proportion of browning (APB) of the FCSPSs after different treatments during storage. (**a**) APB of CK and three LA treatments; (**b**) APB of CK and three LF treatments; (**c**) APB of CK and three LP treatments. Note: Values within the same storage day followed by the different letters are significantly different at the 5% level according to ANOVA–Duncan’s multiple range test.

Visual appearance determines the value of fresh-cut products, usually influencing consumer acceptance. The color of the flesh is one of the most essential criteria for the appearance quality of FCSPSs [27]. In this study, the flesh natural color of Xinxiang was yellow. It is easier to observe the browning spots than in purple flesh cultivars. As shown in Figure 1, we found that the browning area proportion increased as time went on. APB of CK increased sharply at DAS 1 and DAS 5. APB of CK increased by 6.60% from zero at 1 DAS. Three licorice extract treatments had the same trend. Their APB increased to 4.15~8.01% from zero. It is worth noting that the APB of LA30, LA50, LF10, LF50, and LP30 was significantly lower than CK at 1 DAS (4.15~5.99%, *p* < 0.05). It indicated that they have the effect of reducing browning at 1 DAS. Then, we found that the APB of LA and LF did not increase sharply at other storage times. They still kept 4.00~10.50% from 3 to 7 DAS. Meanwhile, the APB of both LA and LF treatments was significantly lower than CK at 5 and 7 DAS (*p* < 0.05). However, LP treatments had a similar trend to CK after 1 DAS. The APB of LP maintained growth during 1 to 7 DAS, although the APB of LP10 was significantly lower than CK during 3 to 7 DAS (6.43~17.02%, *p* < 0.05). Even the APB of LP30 exceeded CK at 3 to 7 DAS. It indicated that LA and LF treatments have a good color protection effect on FPSPS within one week; they inhibit the expansion of the browning area of FPSPS.

**Figure 2:**
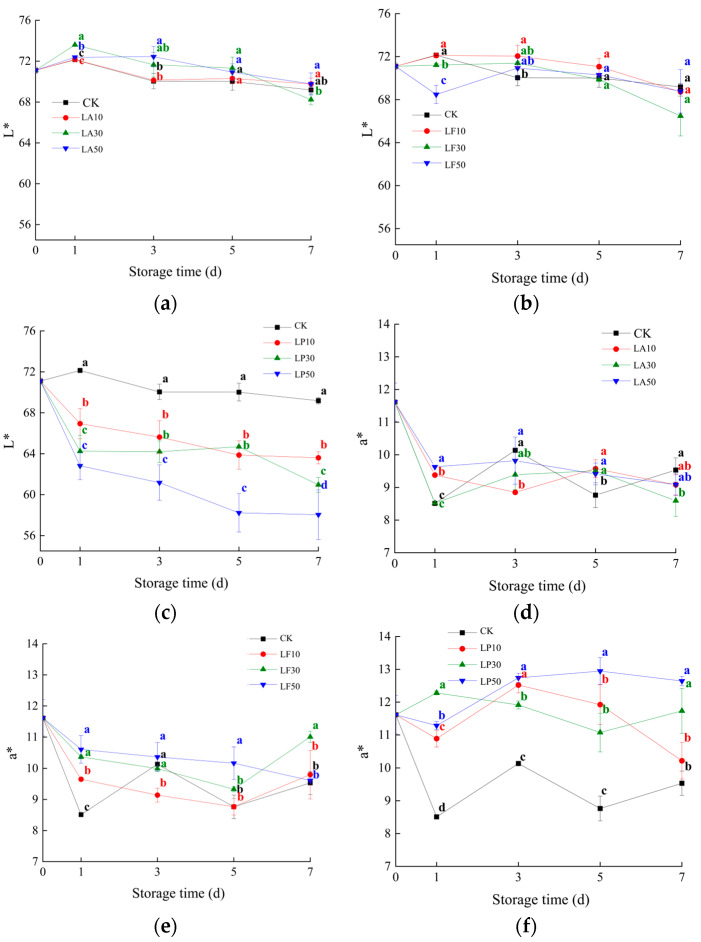

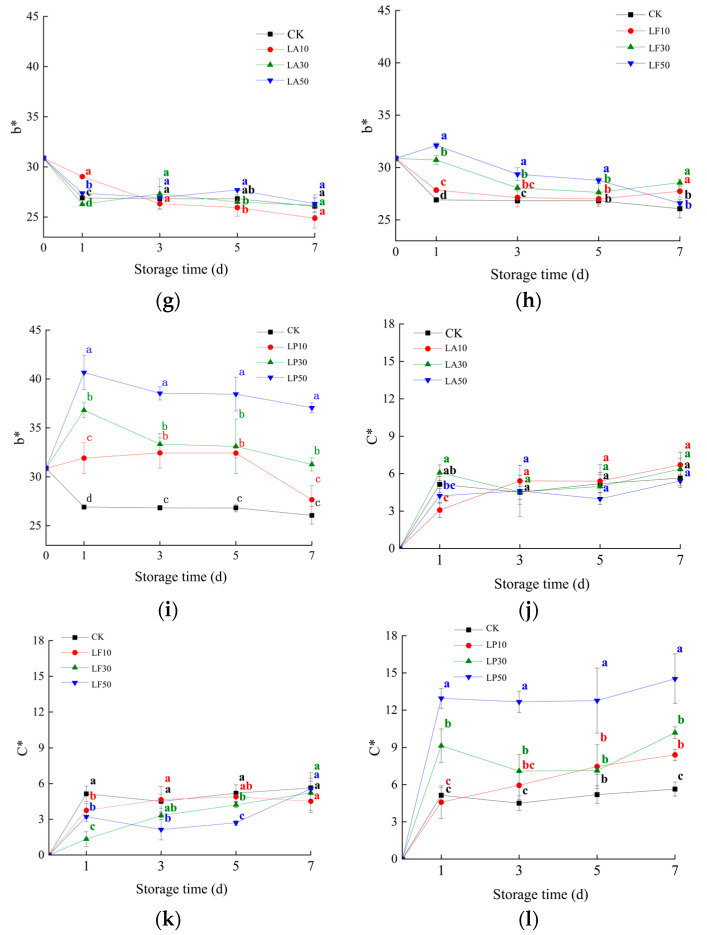
Chromatic coordinates of the FCSPSs after different treatments during storage. (**a**) L* value of CK and three LA treatments; (**b**) L* value of CK and three LF treatments; (**c**) L* value of CK and three LP treatments; (**d**) a* value of CK and three LA treatments; (**e**) a* value of three LF treatments; (**f**) a* value of CK and three LP treatments; (**g**) b* value of CK and three LA treatments; (**h**) b* value of CK and three LF treatments; (**i**) b* value of CK and three LP treatments; (**j**) C* value of CK and three LA treatments; (**k**) C* value of CK and three LF treatments; and (**l**) C* value of CK and three LP treatments. Note: Values within the same storage day followed by the different letters are significantly different at the 5% level according to ANOVA–Duncan’s multiple range test.

As shown in Figure 2, FCSPS of CK was browning obviously at 3 DAS, and then the browning process was accelerated. FCSPS of licorice extract solution was not changed significantly at 3 DAS. FCSPS of LA and LF disappeared many browning spots at 7 DAS; other storage time was not significant. FCSPS of LP disappeared many browning spots at 5 DAS. The L*, a*, and b* values of the FCSPSs decreased during storage in all treatments. The L* values of LF30, LF50, LP10, LP30, and LP50 were significantly lower than CK at 1 DAS (*p* < 0.05), and LA50 and LF10 were higher than CK at 3 DAS (*p* < 0.05). The L* values of LP10, LP30, and LP50 were still lower than CK at any storage time (*p* < 0.05). The a* values of LA10, LA50, LF10, LF50, and both C treatments were significantly higher than CK at 1 DAS (*p* < 0.05), especially the C treatments. They were always higher than CK at any storage time in this study (*p* < 0.05), whereas LA10, LF10, and LF50 were lower than CK at 3 DAS (*p* < 0.05). Both A treatments and LF50 were higher than CK at 5 DAS (*p* < 0.05). LA30 was lower than CK at 5 DAS (*p* < 0.05). The b* values of LA10, LA50, both B treatments, and both C treatments were significantly higher than CK at 1 DAS (*p* < 0.05), and the C treatments were higher than CK at any storage time (*p* < 0.05, except LP10 at 7 DAS), while all A treatments were insignificantly different from CK at 3~7 DAS (*p* > 0.05). LF30 was higher than CK at 1~3 DAS, and LF50 was higher than CK at 1~5 DAS. These results indicate that LP makes the flesh darker, redder, and yellower than CK.

**Figure 3:**
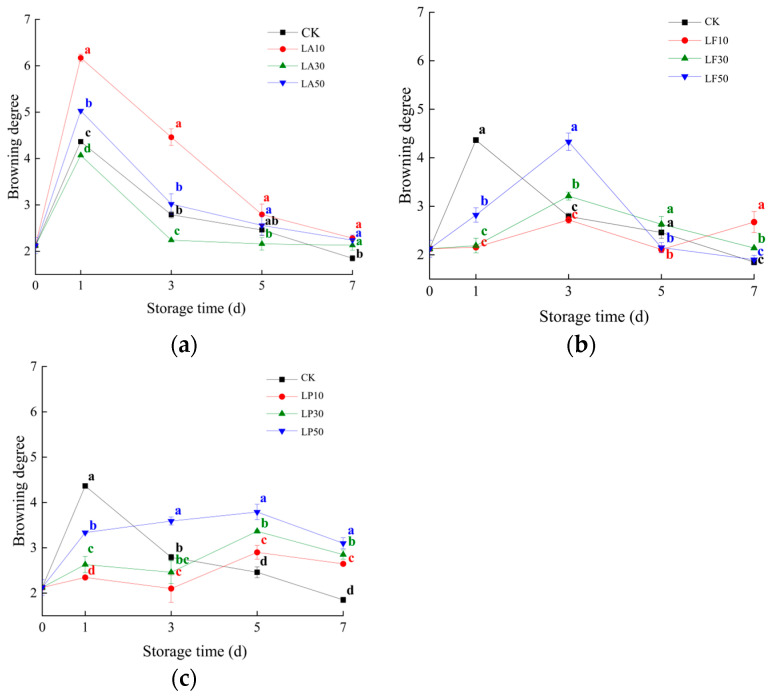
Browning degree of the FCSPSs of licorice extract solution treatments during storage. (**a**) CK and three LA treatments; (**b**) CK and three LF treatments; and (**c**) CK and three LP treatments. Note: Values within the same storage day followed by the different letters are significantly different at the 5% level according to ANOVA–Duncan’s multiple range test.

As shown in Figure 3, we monitored the degree of increased browning between 0 and 7 DAS. CK and Group A grew fast at 1 DAS. The browning degree of LA10 was higher than CK during storage. LA30 was lower than CK during 1~5 DAS. LA50 was lower than CK at 1 and 3 DAS. The assumption is that the LA solution accelerates the browning degree of FCSPSs. The browning degree of Group B was lower than CK at 1 DAS. Then, it increased at 3 DAS and decreased at 5~7 DAS. LF30 and LF50 were significantly higher than CK at 3 DAS (*p* < 0.05), and then LF10 and LF50 were lower than CK at 5 DAS (*p* <0.05). LF10 and LF30 were higher than CK at 7 DAS (*p* < 0.05). The LF solution probably postpones the browning reaction of FCSPSs at 1 DAS; the effect of inhibiting the browning of LF solution was lower than moderate-concentration solutions. The degree of browning in Group C was lower than CK at 1 DAS, and then LP10 was significantly lower than CK (*p* < 0.05). LP50 was significantly higher than CK (*p* < 0.05) at 3 DAS. The degree of browning in Group C was strongly higher than CK during 5~7 DAS. Similar to LF, it is highly likely that the LP solution postpones the browning reaction of FCSPSs at 1 DAS, and high concentrations of LP were not as effective as moderate-concentration solutions. It indicated that 10~30 mg/mL LA, LF, and LP solutions may inhibit the increase in the degree of browning.

### 3.3. Weight Loss Rate and Decay Rate

**Figure 4:**
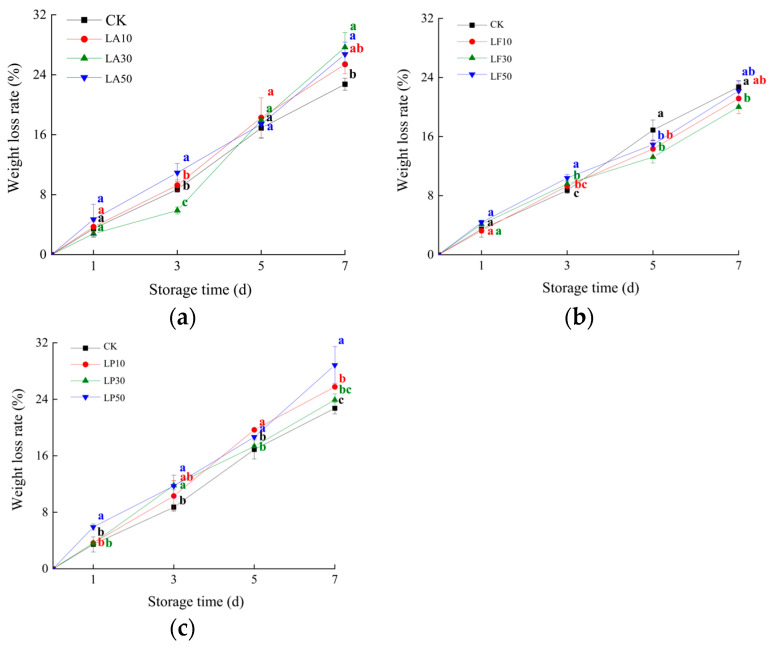
Weight loss rate of FCSPSs after licorice extract solution treatments during storage. (**a**) CK and 3 LA treatments; (**b**) CK and 3 LF treatments; and (**c**) CK and 3 LP treatments. Note: Values within the same storage day followed by the different letters are significantly different at the 5% level according to ANOVA–Duncan’s multiple range test.

We dynamically monitored the weight loss rate (WLR) of FCSPSs in a week (Figure 4). As time goes on, the WLR of FCSPS gradually increases. Only the WLR of LP50 was higher than CK on day 1 (*p* < 0.05); other treatments were not significantly different from CK. On day 3, the WLR of LA30 was 5.89%, significantly lower than CK (8.71%) and other treatments (*p* < 0.05). However, the WLR of LA30 rose rapidly after day 5; it was significantly higher than CK (*p* < 0.05). On days 5 and 7, the WLRs of LF30 were 13.22% and 20.01%, which was significantly lower than CK (*p* < 0.05). These results show that 30 mg/mL LA solution may decrease the WLR of FCSPSs before day 5, and the 30 mg/mL licorice flavonoid solution decreased the WLR of the slices in a week.

**Figure 5:**
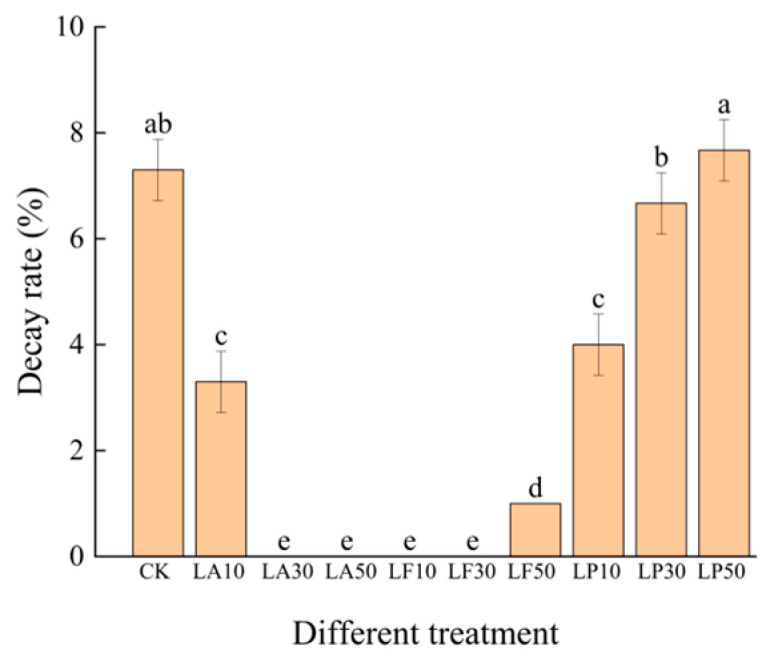
The decay rate of the slices of licorice extract solution treatments during storage at 7 DAS. Note: Values within the same storage day followed by the different letters are significantly different at the 5% level according to ANOVA–Duncan’s multiple range test.

The change in the decay rate (DR) of the FCSPSs at 7 DAS, as Figure 5 shows, the DR of the FCSPSs with all treatments was 0.0% until 5 DAS. As shown in Figure 5, the DR of the FCSPSs with CK was 7.33%. The DR of LA30, LA50, LF10, and LF30 was 0.00%. The DR of LA10 and LF50 was 3.33% and 1.00 %, respectively. They were significantly different from CK (*p* < 0.05). It indicates that moderate LA and LF solutions can effectively inhibit the decay issue in a week. The DR of LP30 and LP50 was not significantly different from CK (*p* > 0.05); it was 6.67% and 7.67%, respectively. However, the DR of LP10 was significantly lower than CK (3.67%, *p* < 0.05). LP may be less or not effective in preventing decay processing on FCSPSs in a week.

### 3.4. Polyphenol Oxidase Activities

**Figure 6:**
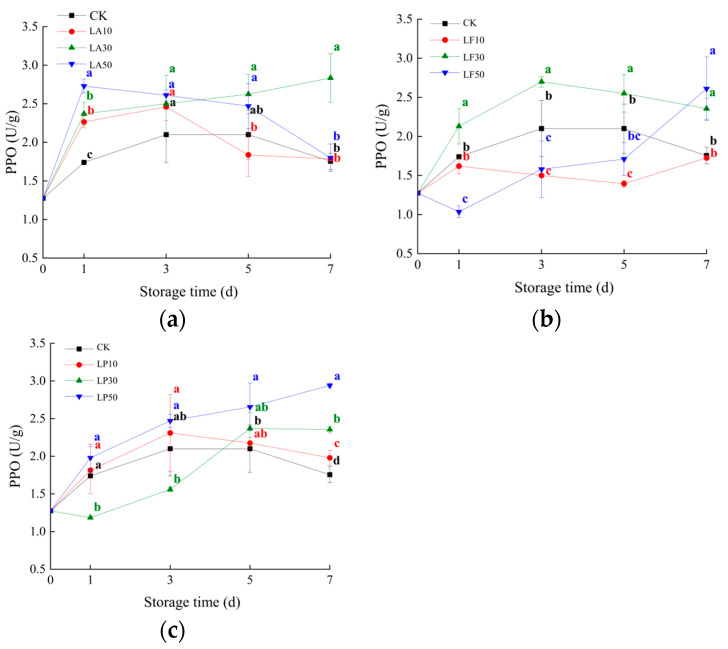
Polyphenol oxidase activities (PPO) activities of the slices of licorice extract solution treatments during storage (**a**) CK and three LA treatments; (**b**) CK and three LF treatments; and (**c**) CK and three LP treatments. Values within the same storage day followed by the same letter are not significantly different at the 5% level according to ANOVA–Duncan’s multiple range test.

Enzyme activity in the antioxidant system is an important indicator that affects and reflects the physiological and metabolic changes of FCSPSs. The enzymatic activity of polyphenol oxidase (PPO) has been associated with the deterioration of food quality due to the alteration of organoleptic and nutritional properties. As shown in Figure 6, the PPO activity of CK ranges from 1.5 to 2.0, and it did not change sharply during the storage time. The PPO activity of Group A was significantly higher than CK at 1 DAS, and it was insignificantly difference from CK at 3~5 DAS. LA30 was significantly higher than CK at 7 DAS. The PPO activity of LF10 was significantly lower than CK at 5 DAS. LF30 was significantly higher than CK in one week, and it was insignificantly different from CK at 3~5 DAS. LF50 was significantly lower than CK at 1 DAS and not significantly with CK at 3 and 5 DAS, but it was higher than CK at 7 DAS. The PPO activity of LP10 and LP50 was insignificantly different from CK at 1~3 DAS, and LP10 remained insignificantly different from CK until 5 DAS, and then it was significantly higher than CK at 7 DAS. LP50 was significantly higher than CK at 5~7 DAS. The PPO of LP30 was significantly lower than CK before 5 DAS, and it was insignificantly different from CK at 5 DAS, and then it was significantly higher than CK at 7 DAS. The results showed that these licorice extracts could not effectively reduce the PPO activity of FCSPSs at 7 DAS, while LP and LF solutions increased the PPO activity of the slices. However, partial treatments were effective in controlling the PPO activity of the slices before day 5, such as a 30 mg/mL LP solution.

### 3.5. Peroxidase Activities

**Figure 7:**
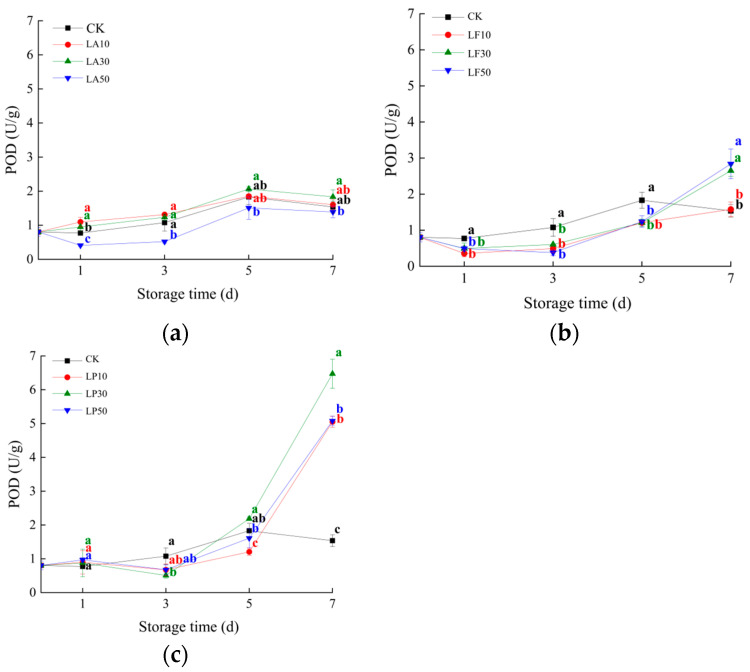
Peroxidase (POD) activities of the slices of licorice extract solution treatments during storage. (**a**) Three licorice acid treatments; (**b**) three licorice flavonoid treatments; (**c**) three licorice polysaccharide treatments. Note: Values within the same storage day followed by the different letters are significantly different at the 5% level according to ANOVA–Duncan’s multiple range test.

Peroxidase (POD) is an oxidoreductase that can perform the single-electron oxidation of phenolic compounds in the presence of hydrogen peroxide. It protects the flesh of sweet potatoes from oxidative damage. As shown in Figure 7, the POD activity of LA10 and LA30 was significantly higher than CK at 1 DAS (*p* < 0.05), and then it was insignificantly different from CK at 3~7 DAS (*p* > 0.05). LA50 was lower than CK significantly at 1 to 3 DAS in this study (*p* < 0.05). The POD activity of LF treatments (LF10, LF30, and LF50) was significantly lower than CK during 0 to 5 DAS, and only LF30 and LF50 were higher than other treatments at 7 DAS (*p* < 0.05). The POD activity of LP treatments was insignificantly different with CK at 1 DAS (*p* > 0.05), and then LP30 was significantly lower than CK at 3 DAS (*p* < 0.05). At 5 DAS, LP10 was significantly lower than CK (*p* < 0.05). It is worth noting that the POD activity of all LP treatments was significantly higher than CK at 7 DAS. This indicated that all licorice extraction solutions were not considerably increased by POD at 1 to 5 DAS. The LA solution was not increased by POD activity in the flesh at any time. In total, 30~50 mg/mL of LF solution and all LP solution treatments made the POD activity of the slices increase at 7 DAS.

### 3.6. Superoxide Dismutase Activities

**Figure 8:**
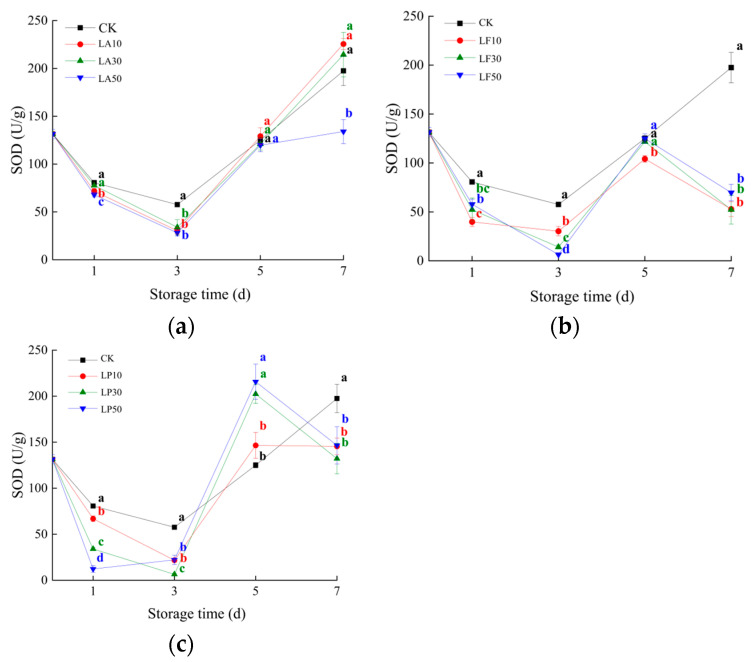
Superoxide dismutase (SOD) activities of the slices of licorice extract solution treatments during storage. (**a**) Three licorice acid treatments; (**b**) three licorice flavonoids treatments; and (**c**) three licorice polysaccharides treatments. Note: Values within the same storage day followed by the different letters are significantly different at the 5% level according to ANOVA–Duncan’s multiple range test.

Superoxide dismutase (SOD) is an essential enzymatic antioxidant found in every plant cell that plays an essential role under various abiotic stress conditions. As shown in Figure 8, the SOD activity was not increased by all licorice extraction treatments during 1 to 3 DAS (*p* > 0.05). Partial treatments even decreased the SOD activity of the slices during 1 to 3 DAS. At 5 DAS, the SOD activity of all LP solution treatments was higher than CK, and the SOD activity of C2 and C3 increased significantly (*p* < 0.05). The SOD activity of other licorice extraction treatments was not higher than CK at 5 DAS. The SOD activity after licorice extract processing was not significantly higher than CK at 7 DAS.

### 3.7. Correlation Analysis

**Figure 9:**
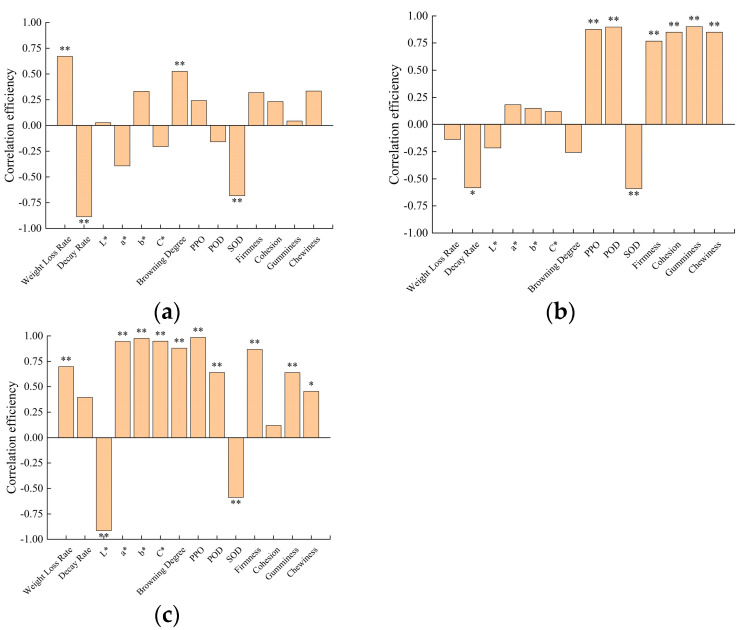
The correlation efficiency of solution concentration to physical and chemical properties of slices at 7 DAS (*n* = 40). (**a**) LA; (**b**) LF; (**c**) LP. Note: * means significant correlation at the 5% level and ** means significant correlation at the 1% level.

We attempted to explore the correlation efficiency between the solution concentration of three licorice extractions and the physical and chemical properties of FCSPSs after 1 week of storage. As Figure 9 shows, the concentration of the LA solution kept a stronger positive correlation with WLR and the degree of browning and kept a stronger negative correlation with decay rate and SOD activity at 7 DAS. The concentration of the LF solution kept a stronger positive correlation with the PPO activity, POD activity, firmness, cohesion, gumminess, and chewiness of FCSPSs, and then it kept a stronger negative correlation with the SOD activity of the FCSPSs at 7 DAS. The concentration of the LP solution kept a stronger positive correlation with the WLR, a* value, b* value, C* value, browning degree, PPO activity, POD activity, firmness, gumminess, and chewiness of the FCSPSs, and then it kept a stronger negative correlation with the L* value and SOD activity of the FCSPSs at 7 DAS. This result indicates that a high concentration of LA solution caused the decay rate to decrease at 7 DAS. High concentrations of the three licorice extract solutions caused the SOD activity of the FCSPSs to decrease. High concentrations of LA and licorice polysaccharide solution promoted an increase in the weight loss rate and degree of browning of the FCSPSs.

### 3.8. Cluster Analysis

**Figure 10:**
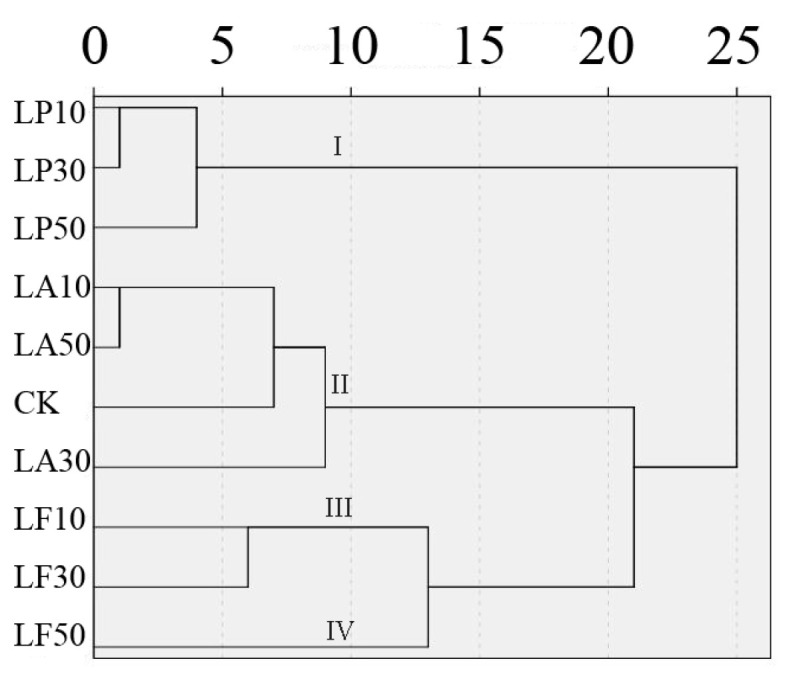
Cluster analysis of FCSPSs quality traits by 10 treatments at 7 DAS. Note: A Euclidean distance of 10 was used to classify the groups.

Cluster analysis is better for classifying different treatments. First, the quality of FCSPSs at DAS 7 was standardized to remove the influence of units, and then cluster analysis was performed based on the inter-group connection and Pearson correlation (Figure 10). Both LP treatments were classified as Group I, both LA treatments and CK were classified as Group II, LF10 and LF30 were classified as Group III, and LF50 was classified as Group IV. The four groups had different characteristics. FCSPSs’ characteristics of Group I were low L* value and chewiness in four groups; meanwhile, it was high a* value, b* value, C* value, browning degree, and PPO and POD activity in four groups. FCSPSs’ characteristics of Group II were high L* value and low a* value, b* values, browning degree, firmness, gumminess, and PPO and POD activity. SOD activity, weight loss rate, and decay rate of Group III were lower than other groups, with moderate texture properties and flesh color. The cohesion, gumminess, and chewiness of Group IV were higher than other groups. It indicated that the composition of licorice extract is more important than the concentration. LA and LF are better than LP as one component of FCSPSs’ additives. Moderate LF concentration treatment is more suitable than other concentration treatments.

## 4. Discussion

### 4.1. The Effect of Licorice Extracts on FCSPSs in Texture Properties

Tuberous roots of sweet potatoes still have metabolic activity after harvest, so their quality and shelf life are important issues, especially during distribution and storage [28]. The treatments during storage make the quality change. Previous studies found the texture properties, flesh color, and moisture content of the tuberous root of sweet potato were changed during storage at 5 °C. The firmness and moisture content, L* value(flesh), of tuberous roots decreased over the storage time, and the cohesion, a* value(flesh), of tuberous roots increased over the storage time at 5 ℃ [29]. We found the firmness, cohesion, and L* value(flesh) of CK’s FCSPSs had a similar trend in this study. The firmness of CK at 7 DAS was lower than at 1 DAS, and the weight loss rate was increasing during storage at 4 °C in this study. However, we found the firmness of FCSPSs was kept stable or increasing after the licorice extract treatments at 1, 5, and 7 DAS in this study. It indicated three licorice extracts impacted keeping or increasing the firmness of tuberous sweet potatoes. Benzothiazole accelerated lignin accumulation in mechanically damaged sweet potatoes, and lignin accumulation increased the firmness of flesh [30,31,32]. So, we assumed the licorice extracts with cutting may have motivated the plant’s immune system and increased lignin and moisture loss, then the firmness of FPSPS increased in this study.

### 4.2. Antioxidant Activity of Licorice Extracts

Previous meat studies found licorice extracts as feed for livestock and food additives for meat can increase the shelf-life time due to improved antioxidant status and protect the meat from lipid oxidation [14,15]. However, licorice extracts used in most previous studies were mixtures. It is hard to confirm which single licorice component affected the shelf life. Meanwhile, the shelf life of fresh-cut fruit and vegetables mostly faced flesh browning issues, not lipid oxidation. The licorice extract treatments changed the flesh color (L*, a*, b* and C*) of FCSPSs during storage. LP treatments decreased the L* of FCSPSs significantly. Moderate LA and LF solutions can inhibit the APB increasing, as this study found. The browning of sweet potatoes is mostly caused by the PPO and POD activity. The enzyme activity of PPO and POD affects the sensory and nutritional characteristics of sweet potatoes, leading to a decrease in their quality. Quinic acids may decrease PPO due to it having an affinity for PPO with caffeic acid [33]. Isoliquiritigenin, isoliquiritin, and licochalcone B had significant scavenging effects on DPPH, ABTS, and FRAP free radicals and displayed good antioxidant activity [18]. However, we found different trends in the three licorice extracts. LA30, LF30, LF50 and all LP treatments increased the enzyme activity of PPO in FCSPSs, and LF30, LF50, and all LP treatments increased the enzyme activity of POD in FCSPSs. To make matters worse, all these licorice extracts treatments reduce the enzyme SOD activity in FCSPSs. Licorice extracts are the addition of naturally potent ingredients that increase the safety of products in the minds of consumers compared to synthetic additives [34,35], such as glycyrrhizinic acid being used as a sweetener, antioxidant, antibacterial agent, flavor enhancer, etc. [36]. Some drink research found LA is a good component to improve the flavor of soft drinks, beer, and other drinks due to it being sweet and healthy [37]. So, we assumed LA in FCSPS is beneficial for improving its flavor because it can increase the sweetness. Early studies have shown that LA and LF had good antimicrobial activity. Low-concentration LA solution (0.03–0.25 mg/mL) inhibited the *Staphylococcus aureus*, *Escherichia coli*, *Bacillus subtilis* and *Bacterium aeruginosa* expend effectively [38,39]. LF inhibited *Staphylococcus aureus*, *usarium graminearum* and *Listeria monocytogenes* expend [40,41,42]. We have found similar results; the decay rate of FCSPS after LA and LF treatments decreased significantly at 7 DAS in this study. High-concentration LA and LF solution treatments can decrease the decay rate of FCSPSs. These results highlight the possible implications of using LA and LF as natural compounds to evaluate their antioxidant efficacy. Notably, understanding the quality of FCSPSs in the presence of LA and LF can help identify the correct use and support application of food additives in the food industry. Composite preservation technology is the research direction for fresh-cut fruits and vegetables in the future [30,43]. Natural preservatives need to be combined with other preservation technologies to be practical. Future studies still need to continue exploring the main components and concentrations of licorice extracts that are effective for FPSPS preservation and developing composite preservatives to improve the quality of FPSPSs during shelf-life storage.

## 5. Conclusions

In conclusion, this study described the texture, appearance quality, and antioxidant capacity of FCSPSs during one week of storage after licorice extract treatments to elucidate the effect of LA, LF, and LP on FCSPS treatments. LA and LF have the potential to be developed as an additive for FCSPSs. They kept the texture properties stable during storage and decreased the decay rate of FCSPSs. The 30 mg/mL LA and LF treatments are good for keeping the appearance and texture quality of FCSPSs during one week of storage. It is worth noting that the PPO and POD activity of FCSPSs after LA and LF treatments were similar to CK, and most licorice extract treatments decreased the SOD activity of FCSPSs in this study. LP accelerated the browning and promoted the PPO and POD activity of FCSPSs. These findings provide important theoretical value for the application of licorice extract in food additives.

## Figures and Tables

**Table 1:** Licorice treatments of fresh-cut sweet potato slices (FCSPSs).

Treatment	Solution	Concentration (mg/mL)
CK	Deionized water	/
LA10	Licorice acid (LA)	10
LA30		30
LA50		50
LF10	Licorice flavonoids (LF)	10
LF30		30
LF50		50
LP10	Licorice polysaccharides (LP)	10
LP30		30
LP50		50

**Table 2:** Effects of three concentration solutions of LA, LF, and LP on the firmness of FCSPSs.

Treatments	Day after Storage (DAS)				
	0	1	3	5	7
CK	78.78 ± 5.28	84.50 ± 5.82 b	75.96 ± 1.58 e	82.41 ± 6.57 de	71.91 ± 5.08 e
LA10		84.24 ± 4.61 b	79.51 ± 3.62 de	86.57 ± 5.71 bcde	87.32 ± 5.81 cd
LA30		85.10 ± 3.92 b	79.23 ± 4.57 de	80.17 ± 5.32 e	82.72 ± 6.62 d
LA50		85.71 ± 2.93 b	86.70 ± 4.37 c	83.67 ± 1.35 cde	84.03 ± 2.01 d
LF10		82.68 ± 5.95 b	88.12 ± 4.01 bc	89.12 ± 3.01 abc	85.91 ± 2.86 cd
LF30		84.53 ± 2.25 b	87.40 ± 2.12 bc	91.55 ± 4.05 ab	88.26 ± 4.32 bcd
LF50		85.08 ± 5.64 b	93.74 ± 3.26 a	93.27 ± 5.87 a	93.54 ± 3.82 b
LP10		83.22 ± 3.40 b	83.84 ± 6.08 cd	84.49 ± 2.89 cde	85.79 ± 4.98 cd
LP30		96.26 ± 3.18 a	84.79 ± 2.41 c	85.66 ± 4.44 bcde	91.48 ± 3.08 bc
LP50		99.08 ± 4.59 a	92.21 ± 5.06 ab	87.06 ± 3.18 abcd	99.24 ± 3.91 a

Note: Values within the same storage day followed by the different letters are significantly different at the 5% level according to ANOVA–Duncan’s multiple range test.

**Table 3:** Effects of three concentration solutions of LA, LF, and LP on the cohesion of FCSPSs.

Treatments	Day after Storage (DAS)				
	0	1	3	5	7
CK	0.15 ± 0.04	0.10 ± 0.02 e	0.11 ± 0.02 d	0.14 ± 0.06 b	0.13 ± 0.02 d
LA10		0.16 ± 0.01 bc	0.14 ± 0.02 cd	0.15 ± 0.03 b	0.18 ± 0.02 b
LA30		0.17 ± 0.01 b	0.14 ± 0.04 bcd	0.16 ± 0.02 b	0.13 ± 0.03 d
LA50		0.16 ± 0.02 b	0.15 ± 0.03 bc	0.15 ± 0.02 b	0.16 ± 0.02 bc
LF10		0.14 ± 0.01 cd	0.20 ± 0.02 a	0.21 ± 0.02 a	0.13 ± 0.02 d
LF30		0.13 ± 0.01 de	0.17 ± 0.01 ab	0.18 ± 0.03 ab	0.15 ± 0.01 cd
LF50		0.12 ± 0.02 de	0.17 ± 0.02 abc	0.18 ± 0.01 ab	0.29 ± 0.03 a
LP10		0.20 ± 0.03 a	0.20 ± 0.05 a	0.15 ± 0.03 b	0.13 ± 0.02 d
LP30		0.18 ± 0.02 ab	0.17 ± 0.02 abc	0.16 ± 0.03 b	0.13 ± 0.01 d
LP50		0.17 ± 0.02 b	0.20 ± 0.02 a	0.17 ± 0.02 b	0.13 ± 0.01 d

Note: Values within the same storage day followed by the different letters are significantly different at the 5% level according to ANOVA–Duncan’s multiple range test.

**Table 4:** Effects of three concentration solutions of LA, LF, and LP on the gumminess of FCSPSs.

Treatments	Day after Storage (DAS)				
	0	1	3	5	7
CK	9.53 ± 0.52	8.39 ± 1.79 g	9.44 ± 1.04 c	9.87 ± 1.80 e	8.75 ± 4.49 f
LA10		13.22 ± 0.82 cd	10.58 ± 2.09 c	15.29 ± 1.82 bc	21.03 ± 2.48 b
LA30		14.26 ± 1.03 bc	9.42 ± 1.69 c	12.85 ± 1.88 cd	9.69 ± 1.66 ef
LA50		13.09 ± 1.22 cde	11.62 ± 1.80 c	12.82 ± 2.00 cd	13.51 ± 1.62 c
LF10		11.46 ± 0.87 ef	16.24 ± 1.68 ab	18.41 ± 1.98 a	10.55 ± 1.54 ef
LF30		10.59 ± 0.97 f	15.23 ± 1.31 b	15.74 ± 2.01 b	12.94 ± 0.98 cd
LF50		11.68 ± 1.67 def	15.95 ± 1.78 ab	16.37 ± 1.42 ab	26.33 ± 1.81 a
LP10		15.12 ± 1.19 ab	15.16 ± 1.63 b	11.52 ± 2.07 de	11.38 ± 1.93 cde
LP30		16.74 ± 1.74 a	14.32 ± 1.89 b	11.99 ± 1.43 de	11.09 ± 1.58 de
LP50		15.91 ± 1.48 ab	18.10 ± 1.33 a	14.85 ± 1.32 bc	13.26 ± 1.25 cd

Note: Values within the same storage day followed by the different letters are significantly different at the 5% level according to ANOVA–Duncan’s multiple range test.

**Table 5:** Effects of three concentration solutions of LA, LF, and LP on the chewiness of FCSPSs.

Treatments	Day after Storage (DAS)				
	0	1	3	5	7
CK	52.40 ± 4.90	48.16 ± 3.53 ef	59.74 ± 3.72 f	56.55 ± 4.83 cde	55.64 ± 4.49 e
LA10		53.00 ± 2.07 de	44.18 ± 3.06 h	57.62 ± 3.32 cd	65.19 ± 3.38 b
LA30		65.13 ± 4.29 c	54.92 ± 2.71 g	57.72 ± 4.18 cd	57.72 ± 3.23 de
LA50		70.06 ± 4.27 c	65.06 ± 3.91 de	55.67 ± 2.57 de	64.12 ± 4.19 bc
LF10		46.43 ± 4.67 f	62.21 ± 4.75 ef	64.81 ± 3.04 b	53.86 ± 3.10 e
LF30		57.12 ± 4.81 d	69.11 ± 2.04 d	78.30 ± 3.86 a	63.00 ± 4.61 bcd
LF50		67.70 ± 2.88 c	83.86 ± 3.89 b	79.10 ± 4.44 a	95.23 ± 1.03 a
LP10		105.83 ± 4.01 a	85.91 ± 3.42 b	51.58 ± 2.98 e	45.27 ± 4.35 f
LP30		78.51 ± 4.63 b	74.64 ± 4.57 c	59.74 ± 4.72 bcd	57.40 ± 4.56 de
LP50		82.22 ± 4.67 b	100.39 ± 3.39 a	61.93 ± 4.13 bc	58.69 ± 4.76 cde

Note: Values within the same storage day followed by the different letters are significantly different at the 5% level according to ANOVA–Duncan’s multiple range test.

## Data Availability

Data is contained within the article and the Supplementary Materials.

## Supplementary Materials

The following supporting information can be downloaded at: https://www.mdpi.com/article/10.3390/foods13020211/s1, Figure S1. FCSPSs by different licorice extract solution treatments.
