# Supplementary figures and images for: Three Licorice Extracts’ Impact on the Quality of Fresh-Cut Sweet Potato (Ipomoea batatas (L.) Lam) Slices
Source: Foods. 2024 Jan 9;13(2):211. doi: 10.3390/foods13020211 (PMC10815067; doi:10.3390/foods13020211)

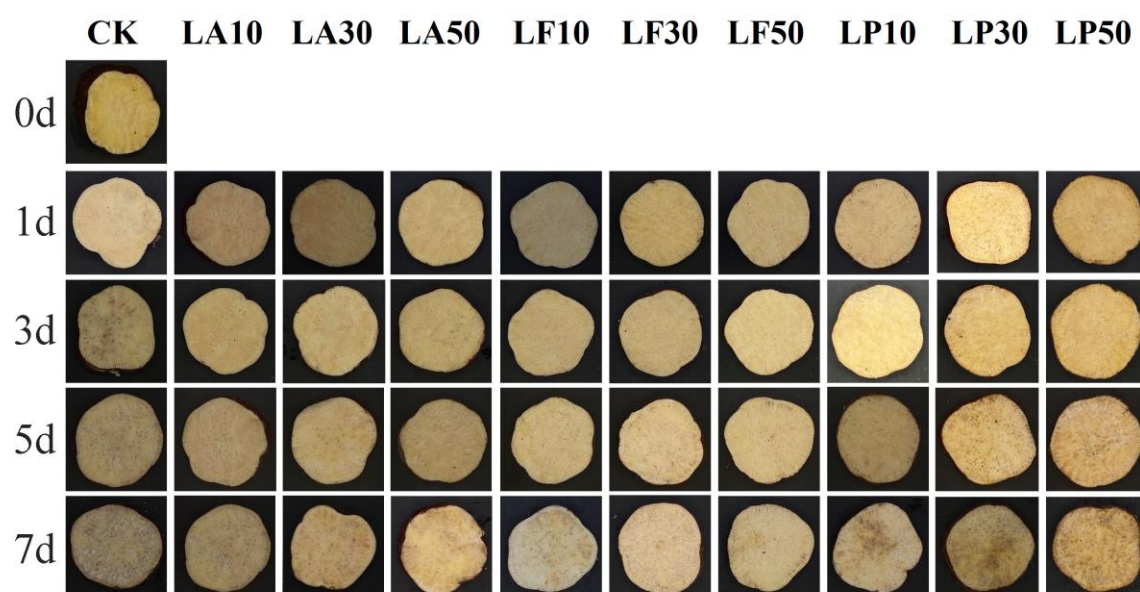

**Figure S1.** FCSPSs by different licorice extract solution treatments.

Supplement: Supplementary file 1 [file foods-13-00211-s001.zip › foods-2785996-supplementary.pdf]
